# Antielastase Activity of Macassar Kernels (*Rhus javanica)* Stem Extract and Skin Elasticity Evaluation of Its Topical Gel Formulation

**DOI:** 10.1155/2021/6690029

**Published:** 2021-04-21

**Authors:** Nadia Bunga Anggraini, Berna Elya, Iskandarsyah Iskandarsyah

**Affiliations:** Faculty of Pharmacy, Universitas Indonesia, Depok, Indonesia

## Abstract

**Background:**

Macassar kernels (*Rhus javanica* L.) has potential as an antiaging agent as it has antielastase activity, especially its stem extract which has best percent inhibition compared to its leaves and fruit extract. Moreover, the antiaging agent can be commonly used in the form of gel for topical applications. Hence, formulation of HEC-based topical gel from the stem extract of Macassar kernels was conducted. This study aims to determine the antielastase activity of the stem extract of Macassar kernels and evaluate the skin elasticity of its topical gel formulation by conducting dermatological safety and skin antiaging efficacy test.

**Methods:**

The stem extract was in vitro tested for antielastase activity using a microplate reader. Then, a formulation of a topical gel containing *Rhus javanica* stem extract was made. Five stages of quality control, which consisted of an organoleptic test, homogeneity test, pH measurement, viscosity measurement, and physicochemical stability test, were conducted to ensure the quality of topical gel formulation. Last, clinical studies were conducted to evaluate the dermatological safety and antiaging efficacy of gel preparation containing stem extract of *Rhus javanica. Results*. The stem extract provided antielastase activity (IC50 = 245.68 *μ*g/mL), and its polyphenol was valued at 23.28 ± 1.52 mg GAE/g). The gel containing 10% stem extract had better stability than the gel containing 5% stem extract. The dermatology safety test and efficacy test results indicated that the topical gel containing 10% *Rhus javanica* stem extract did not cause any skin irritation and significantly improved skin elasticity (*p* < 0.05). In the treatment group, the moisture parameter was significantly changed on day 14 (*p* < 0.0001), day 21 (*p* < 0.0001), and day 29 (*p* < 0.0001). The elasticity parameter was also changed significantly on day 14 (*p*=0.0485), day 21 (*p*=0.0537), and day 29 (*p*=0.0002).

**Conclusion:**

The stem extract of *Rhus javanica* has potential antielastase activity. The topical gel containing *Rhus javanica* stem extract also has potential antielastase activity by increasing the skin moisture and enhancing skin elasticity.

## 1. Introduction

Aging is a complex biological process causing morphological and physiological changes. The aging process of the skin occurs progressively, especially in the skin layer exposed to sunlight [1]. The characteristic of skin aging is marked by the appearance of distinctive cutaneous signs in the form of wrinkles and an increased level of sagging skin [2].

Based on data from the United Nations [3], the elderly population (over 65 years old) is 703 million and is expected to double by 2050. In Indonesia, the elderly population had doubled over nearly half a century (1971–2019). In 2019, the elderly population in Indonesia reached 9.6% of about 25.64 million people. A country would become an aging population if its elderly population were more than 10% [4]. This situation reflected Indonesia's growing life expectancy, although health problems related to skin aging increased.

Many cosmetics contain synthetic materials that react disadvantageously to the skin. Therefore, researches on the potential of herbal material as a cosmetic agent are increasing significantly. Secondary metabolite found in plants had been widely proven to have skin protection activity, both in vitro and in vivo [5]. Some phenolic compounds, such as flavonoids, stilbenes, and hydroxycinnamic acid derivatives, have the potential activity to decrease the production of Reactive Oxygen Species (ROS). This potency was closely related to the protective function of polyphenol compounds in inhibiting elastin degradation [6].

*Rhus javanica,* a native plant from Indonesia, can be an alternative to produce cosmetic active ingredients. Its potential as an antiaging agent has been proven by Moon et.al.[7]. In the previous research, stem extract of *Rhus javanica* had the best percent inhibition of elastase activity compared to extract from leaves and fruits (greenish and blackish-grey) [8].

Topical applications of antiaging cosmetics can be commonly used in the form of a gel due to the superiority of gel form characteristics, as it is not sticky, dries easily, and forms a thin film layer that can be easily cleaned. Hydroxyethyl cellulose (HEC) can be used as a gel base because it has a pH that is not too acidic, is hygroscopic, and has the viscosity and dispersive power required in topical gel preparations. The pH characteristic of hydroxyethyl cellulose, which is not too acidic (between 5.5 and 8), is expected to maintain the quality of phenolic compounds in the extract [9].

Based on the above matters, this study investigated antielastase activity as the antiaging properties of Macassar kernels (*Rhus javanica*) stem extract. This research was conducted in vitro to determine the IC50 value. Furthermore, formulation of HEC-based topical gel from the stem extract of *Rhus javanica* was conducted. To ensure the safety of the topical gel, a dermatological safety test (skin irritation evaluation) of the selected formulation was done by assessing the presence of erythema and edema as the good topical gel should not have an irritant effect. The skin elasticity evaluation of the topical gel formulation was then conducted to determine the antiaging efficacy of the topical gel formulation by checking the skin moisture and elasticity level using a skin analyzer.

## 2. Materials and Methods

### 2.1. Materials and Chemicals

*Trizma Base*, *N-Succinyl-Ala-Ala-Ala-p-nitroanilide*/SANA, *porcine pancreas elastase*, and gallic acid were purchased from Sigma Aldrich (Germany). Hydrochloric acid, dimethyl sulfoxide, ethanol and methanol, Folin–Ciocalteu reagent, and sodium carbonate were purchased from Merck (Germany). Glycerine, propylene glycol, and edetate sodium were purchased from Amresco Chemicals (China). Hydroxyethyl cellulose was purchased from Fracxchange (USA). Propylparaben and methylparaben were purchased from Gujarat Organic (India). Distilled water was purchased from Brataco Chemika (Indonesia).

### 2.2. Collection of Plant Materials

*Rhus javanica* was obtained from Pananjung Pangandaran Nature Reserve, West Java, Indonesia, at an altitude of 100 meters above sea level. The stem of *Rhus javanica* was determined (No. 3912/11.CO2.2/PL/2019) at the School of Life Science and Technology, Bandung Institute of Technology, Indonesia.

### 2.3. Extraction

The stem of *Rhus javanica* was dried at 50°C until a stable weight was gained. The samples were ground by a blender to obtain a fine powder. Powdered materials were extracted through maceration method using a distilled solvent. The maceration process was conducted using 96% ethanol with a ratio of 1 : 10. Each sample was extracted for 3 days, filtered every 24 hours, and then evaporated at 40°C with rotavapor and water bath until dry.

### 2.4. Antielastase Activity Test

Antielastase activity test was conducted according to the study by Wittenauer et al.[10] and Popoola et.al.[11]. 20 *μ*L of stem extract diluted in DMSO, 130 *μ*L buffer of Tris-HCl pH 8.0, and 25 *μ*L of porcine pancreas elastase enzyme were incubated on 96-well microplate for 15 minutes at 25°C temperature. The reaction began with the addition of 20 *μ*L SANA substrate after 15 minutes at 401 nm. The final concentration obtained was 0.022 units, PPE enzyme solution of 0.022 units, 0.29 mM SANA substrate, and 150 *μ*g/mL stem extract. Epigallocatechin gallate was used as a positive control. Antielastase activity was assessed in percent inhibition (%) using the following formula:

(1) $$\begin{array}{c} \text{inhibition }\left(\%\right)=\frac{\left(A-B\right)}{A}\;\times 100\%. \end{array}$$

### 2.5. Determination of Total Phenolic Content

Determination of total phenolic content was evaluated using microplate reader instruments according to the Farasat Method [12]. 20 *μ*L of stem extract diluted in methanol and 100 *μ*L of FCR solution were shaken for 1 minute in a 96-well microplate. This mixture was incubated for 5 minutes at room temperature and then 80 *μ*L sodium carbonates was added. The mixture was then shaken for 1 minute and incubated for 120 minutes at room temperature. The absorbance value was measured at 720 nm. The calculation of total phenolic content was converted using curve calibration of gallic acid (4–24 *μ*g/mL).

### 2.6. Phytochemical Screening and Nonspecific Parameter Test

In this study, phytochemical screening and nonspecific parameter tests were conducted. The phytochemical screening tests were conducted to detect flavonoid through Clemmensen reduction using Zn and HCl, phenol using Folin–Ciocalteu assay, saponin using foam test, tannin using gelatine and gelatine-salt test, and terpenoid using Liebermann–Burchard reagent. The nonspecific parameter tests were conducted to determine total ash value and acid insoluble ash value.

### 2.7. Formulation of Topical Gel Containing *Rhus javanica* Stem Extract

In the first step, 70°C water was poured into the first beaker glass and then sprinkled with hydroxyethyl cellulose. It was kept in the beaker to swell for 10 minutes and then stirred at 1200 rpm for 30 min until it formed a gel perfectly (A). Then, in the second beaker glass, methylparaben and propylparaben were added to propylene glycol while being stirred and subsequently added with glycerin until dissolved and clear (B). In the third beaker glass, Na-EDTA was dissolved in 20% heated water (C). Then, B and C were poured into the gel base (A) and stirred with a medium-speed homogenizer until dispersed. In the final stage, the stem extract of *Rhus javanica* was dissolved in ethanol, then poured into the gel base (mixture of A, B, and C), and stirred with a low-speed homogenizer until completely mixed. Last, the gel was stored at room temperature in a tube.

Formulation of the topical gel containing *Rhus javanica* stem extract can be seen in Table 1. There were two types of topical gel formulation which differed in terms of the active ingredient concentration, i.e., F1 with 5% *Rhus javanica* stem extract and F2 with 10% *Rhus javanica* stem extract. In this research, the basis gel was referred to as F0.

### 2.8. Quality Control of Topical Gel Formulation

Five stages of quality control were conducted to ensure the quality of topical gel formulation: organoleptic test, homogeneity test, pH measurement, viscosity measurement, and physicochemical stability test.

#### 2.8.1. Organoleptic Test

An organoleptic test was carried out visually by directly paying attention to the shape, color, and smell of the gel containing *Rhus javanica* stem extract. Color criteria were evaluated based on the Parameters of Color Standards and Color Nomenclature [13]. The gels are usually clear, homogeneous, and semisolid preparations [14].

#### 2.8.2. Homogeneity Test

Homogeneity was evaluated by tactile perception. 0.5 grams of gel was applied on a glass plate, then rubbed, and touched.

#### 2.8.3. pH Measurement

The pH measurement of the gel was carried out using a digital pH meter by dunking the glass electrode completely into the gel system to cover the electrode. The measurement was evaluated in triplicate and the average pH value was reported.

#### 2.8.4. Viscosity Measurement

Gel viscosity was measured using Brookfield LV viscometer. This was done by dunking the spindle into the gel preparation. The speed of viscometer was set at 0, 5, 2, 5, 10, then 20 rpm, then the reverse round from 20, 10, 5, 2, 5, to 0 rpm. Reading dial scale (dr) was performed when the red needle movement has stabilized. The viscosity value was derived from the multiplication of the correction factor (*f*) and the reading dial (dr), calculated from each speed value. Gel viscosity measurements were conducted at week 0 and week 12.

#### 2.8.5. Physicochemical Stability Tests

Physicochemical stability tests of the topical gel were conducted by keeping the topical gel for 12 weeks at 3 levels of temperature, i.e., low temperature (4 ± 2°C), room temperature (27 ± 2°C), and high temperature (40 ± 2°C). The physicochemical stability tests, including evaluation of color, odor, pH, viscosity, phenolic content, and freeze-thaw cycle test, were conducted every 2 weeks or 14 days in 6 cycles. Hence, the physicochemical stability of the topical gel was evaluated at days 0, 14, 28, 42, 56, 70, and 84.

### 2.9. Clinical Studies

Clinical studies were conducted as a preliminary study that evaluated the dermatological safety and antiaging efficacy of gel preparation containing stem extract of *Rhus javanica,* which had antielastase potentiality, to volunteers. The research has passed an ethics review by the ethics committee of the Faculty of Medicine, Universitas Indonesia, No. KET913/UN2. F1/ETHICS/PPM.00.02/2019. All volunteers signed written informed consent. The double-blind randomized controlled trial was chosen to prevent bias in the results of the study. About 30 volunteers were divided into two groups: the treatment group and the placebo group.

The study was conducted on 30 healthy female volunteers between the ages of 19 and 35 years. This study excluded people with the following condition criteria as volunteers: (1) having atopic dermatitis and bronchial asthma history, (2) having chronic dermatitis history (psoriasis and SLE), (3) having a chronic systemic disease (hypertension, diabetes mellitus, chronic kidney failure, and cancer), (4) taking topical or systemic medication and supplements for skin treatment over past four weeks; (5) pregnancy and lactation. The volunteers that passed the dermatological safety test continued to do a clinical trial. Each volunteer filled in questionnaire data regarding their health status, received physical examinations conducted by researchers, and filled in data on the daily use of topical gel and complaints that might arise during the study.

#### 2.9.1. Dermatological Safety Test (Skin Irritation Evaluation)

Dermatological safety test was carried out with Finn chamber patch test. The methods were based on Colipa Guidelines [15] and Tardiff Scale [16]. About 0.2 Gram of topical gel was applied on the chamber. The sample weight was fixed to avoid sliding of the gel and easily distinguish skin irritation caused by the materials chamber.

The volunteers were attached with Finn chamber on the volar area of the upper arm, gel base on the right side, and gel containing extract on the left side. The skin area should be free from dense hair nevus, tattoos, and cosmetics. All volunteers were prohibited from overactivity and contact with water during testing to avoid patch testers from detaching. The volar upper arm was chosen in this study because the forehead and volar forearm area were widely exposed to water where all Muslim participants perform ablution 5 times a day.

The patch tester material was removed after 24 hours and the reaction was evaluated 15 to 20 minutes after tape removal. The interpretation of patch test reaction can be seen in Table 2 of the Tardiff Scale. The reaction of the patch test would be considered as a skin irritant only when the score was equal to or above ‘3', which was characterized by emergence of definite edema (3), definite edema dan papules (4), or vesicular/bulbous eruption.

#### 2.9.2. Antiaging Efficacy Test (Skin Analyzing Methods)

The antiaging efficacy tests were conducted by measuring the level of elasticity and humidity using the EH 900 U skin analyzer. The measurement results were interpreted based on the elasticity and moisture parameters in Table 3. The measurements were taken five times in 4 weeks, i.e., before testing on day 1 (T0), day 7, day 14, day 21, and day 29 (a day after the last day of gel usage). About ten minutes before measurement, the skin area was cleaned with an alcohol pad. The skin area was applied with 0.2 grams of gel or equivalent to 1 Fingertip Unit (FTU), a reference for dermatologists on how much topical preparation should be given on the human body [17]. The gel was applied twice a day, in the morning after shower and before bedtime, for 28 days. To facilitate the measurement, each volunteer was given 1 tube of gel (Netto 20 Gram).

### 2.10. Statistical Analysis

One-way analysis of variance (ANOVA) and Kruskal–Wallis tests were used to obtain eventual variation between different time intervals of efficacy test results. Using these methods, the relationship between duration of topical gel usage and skin elasticity level and the relationship between duration of topical gel usage and skin moisture level were evaluated. The analysis was conducted of each kind of efficacy test treatments, i.e., placebo (gel-based F0) and treatment (gel with *Rhus javanica* stem extract).

Independent *t*-test was used to evaluate whether there was a statistically significant difference between the efficacy test results caused by different types of gel in the placebo group and treatment group. The one-tailed hypothesis *t*-test was used to see whether the treatment group possessed higher efficacy than the placebo group. These tests were conducted to evaluate the difference in skin moisture level improvement caused by different types of gel and the difference in skin elasticity level improvement caused by different types of gel. The significant differences of skin moisture level improvement and the significant differences of skin moisture level improvement caused by different types of gel were evaluated at each measurement (after 7 days of usage, after 14 days of usage, after 21 days of usage, and after 29 days of usage). The skin elasticity and moisture level improvement were calculated based on the percent change of the condition on day 1 (T0).

Pearson's correlation was used to evaluate the relationship between the in vitro antielastase activity and efficacy test. The test analyzed the correlation of IC50 antielastase and elasticity level and also the correlation of IC50 antielastase and moisture level. In this study, a statistically significant difference was considered at *p* value < 0.05 and *p* value < 0.10.

## 3. Results and Discussion

### 3.1. Antielastase Activity Essay

The antielastase activity was done by assessing the difference in absorbance obtained from the sample and control test. Epigallocatechin gallate (EGCG) was used as a positive control. EGCG was widely used as a positive control in previous studies related to the antielastase activity from natural plants [18]. According to the research conducted by German-Bae et al. [19], EGCG has an IC_50_ value of 61.1 *μ*g/mL. There was also another study mentioning the IC_50_ of EGCG at 93.99 ± 3.44 *μ*g/mL [20]. The IC_50_ of EGCG in this study was 57.43 *μ*g/mL. The result was derived from the regression equation *y* = 0.4408*x* + 24.686 with a correlation value of *r* = 0.9958. These values were slightly different from previous studies. This could be due to the different testing conditions. The results of the antielastase activity of EGCG can be seen in Table 4.

Regarding antielastase activity, the IC_50_ value of the stem extract was 245.68 *μ*g/mL. The result was derived from the regression equation *y* = 0.1702*x* + 8.186 with correlation value *r* = 0.9989 (Table 5). The potentiality of *Rhus javanica* stem extract in antielastase activity was not as strong as a positive control. However, the IC_50_ of stem extract was four times greater than EGCG. This could be possible because the stem extract was not a pure chemical substance such as EGCG. It was necessary to prove the efficacy of whether the stem extract had the potential in improving skin elasticity.

### 3.2. Total Phenolic Content

**Figure 1:**
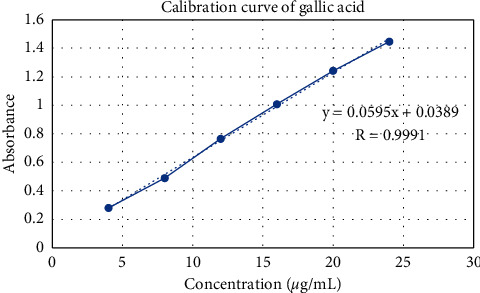
Calibration curve of gallic acid for TPC

Gallic acid was used as the standard for calculating total phenolic content (TPC). TPC resulting in gallic acid equivalents per gram of extract (GAE/g) was extrapolated from a calibration curve of gallic acid (4–24 *μ*g/mL). Gallic acid was diluted into six concentration series and obtained linear regression equations *y* = 0.0595*x* + 0.0389 and correlation coefficient value (*r*) 0.9991. The absorbance obtained from each concentration forms a linear regression curve that can be seen in Figure 1.

The TPC in stem extract was 23.28 ± 1.52 mg GAE/1 g extract. It was obtained from the gallic acid calibration curve. The factors that influenced the phenolic content were genetics, light, temperature, dryness, and salinity [21].

### 3.3. Phytochemical Content

Phytochemical screening can be proven by looking at the discoloration that occurs if the extract was dripped in a particular reagent according to the identified compound. The result showed that the stem extract compounds contained flavonoid, phenol, and terpenoid (Table 6). Flavonoid, phenol, and terpenoid compounds were previously suggested as elastase inhibitors [18].

### 3.4. Nonspecific Parameters

In this research, the total ash value and the acid insoluble ash value were determined. Based on the result, the total ash value was 2.6% and the acid insoluble ash value was 0.02%, as seen in Tables 7 and 8 . The values were eligible and fulfilled the standard of materia medica in Indonesia [22]. These were possible because Pananjung Pangandaran Nature Tourism Park was still maintained, and its location was far from the industrial center.

### 3.5. Gel Preparation Evaluation

**Figure 2:**
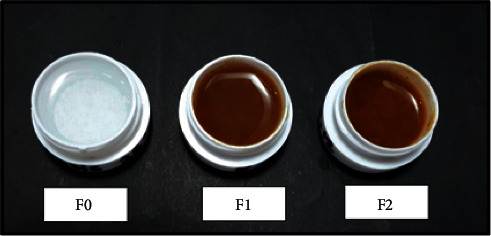
The stability test week- 0.

The organoleptic properties of the topical gel can be seen in Table 9. According to Ridgway's color category (1912), the basis gel F0 had translucent color, whereas F1 and F2 formulas had Brussels brown color (Figure 2). Furthermore, all three formulas were homogeneous, indicating the absence of coarse granules on the object glass.

The pH values of F0, F1, F2, and *Rhus javanica stem extract* were 6.92, 4.88, 4.63, and 4.03, respectively. The addition of stem extract in the gel formulation resulted in a decrease in pH value because of the phenolic compounds in stem extract, which were known to have hydroxyl groups. However, it can be concluded that all formulations were suitable for the human skin pH range (4.5–6.5).

Viscosity was measured using Brookfield viscometer spindle 5 at 20 RPM. The measurement was taken in week 0. The viscosity of F0, F1, and F2 was 30200 cps, 30000 cps, and 22400 cps, respectively. The gel will be more dilute with the addition of the stem extract concentration due to the acidic pH causing hydrolysis of polysaccharide polymer chains into small molecules [23].

**Figure 3:**
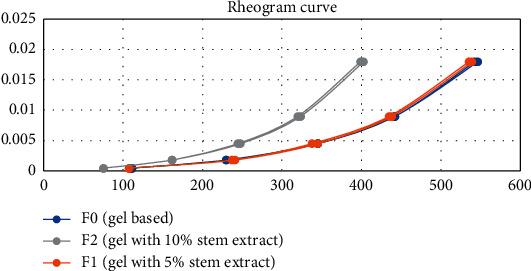
Rheogram curve.

Rheogram of all formulations illustrates the viscosity of thixotropic plastic flow (Figure 3). It corresponds to ideal semisolid preparation, which possesses a thixotropic plastic flow. The plastic flow curve does not pass through the origin (0.0) but cuts the shearing stress axis at one point, namely, the yield value. Thixotropic showed high consistency in the container but will be easily liquid when shaken and easy to spread.

### 3.6. Physicochemical Stability Result

**Figure 4:**
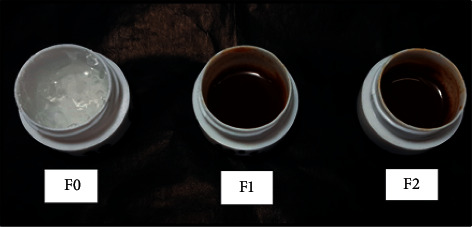
The stability test at week 6 in high temperature.

All preparations stored at low temperature and room temperature did not show discoloration until week 12. It can be concluded that the preparations stored at low and room temperatures were stable. However, the preparations of F1 and F2 stored at high temperature showed a discoloration in which the color turned darker (into raw umber) since the storage period of week 6 (Figure 4). The discoloration was caused by the oxidation of phenolic compound, which formed a darker-colored quinone. In the gel formulation, antioxidant excipients were intentionally not provided. The aim was to determine the antioxidant activity in the stem extract and see its efficacy so that the observation was not biased.

The pH value of F0 during 12 weeks of storage in Table 10 showed that the gel base was fairly stable, especially in low and room temperature storage. The change of gel base pH value was still within the range of the skin pH. At low temperatures, the pH value changed by 0.41 units from 6.92 to 6.51. At room temperature, the pH value changed by 1.11 units from 6.92 to 5.81. Therefore, hydroxyethyl cellulose (HEC) as a gelling agent has good acceptability with natural active ingredients, especially those with phenolic compounds. HEC was stable in a fairly wide pH range of 2–12 [9]. Meanwhile, the pH value of both F1 and F2 decreased starting from week 2 to week 12. In week 10 at high-temperature storage, the preparations could no longer be measured because of syneresis. The decrease in the pH value of F1 and F2 was caused by phenolic compounds coming out of the gel matrix, which increased the acidity of the preparations. When it was associated with organoleptic evaluation, the results were directly proportional to the discoloration, especially at high-temperature storage. The longer the preparation was stored, the darker the color was.

The viscosity of F0, F1, and F2 decreased by 0.66%, 1.33%, and 0.89%, respectively, during 12 weeks of stability evaluation. The decrease in viscosity occurred along with the decrease in pH of the gel preparations.

TPC of F1and F2 in week 0 were 110.59% and 81.38% from TPC extract's value. Analysis of phenolic levels in topical gels can be seen in Table 11. At low temperature in week 8 of storage, the TPC of F1 had the lowest value, decreased 17.28%, while F2 was slightly declined (4.65%). At room temperature storage, TPC of F2 tended to be stable (increased 5.15%), compared to that of F1, which decreased by 11.14%. A greater decrease in F1 was likely to be insoluble extract in the gel base. There was maybe a substitute reservoir on the F2 compared to F1. At high-temperature storage, the TPC of F1 and F2 had doubled. The longer the preparation was heated, the more the phenolic compounds were reduced to be molybdenum complex. The formation of the molybdenum complex caused an increase in the absorbance at the microplate reader.

The observations made during the freeze-thaw cycle test were color, odor, and absence of syneresis. After the freeze-thaw cycle test, the results showed that all formulations were stable, characterized by the absence of syneresis. The absence of syneresis was identified by the absence of water found on the upper surface of the gel. All gel preparations also did not experience change in color and odor.

### 3.7. Clinical Studies

In the clinical studies consisting of dermatological safety and efficacy test, the gel base (F0) was used as placebo and gel preparation with 10% stem extract of *Rhus javanica* (F2) was used as treatment. The gel preparation with 10% stem extract was chosen as the treatment because it had organoleptic stability, homogeneity, pH stability, viscosity stability and levels of phenol compounds that still last until week 12, except at high-temperature storage of 40°C. The F1 gel containing 10% stem extract had better stability than F2 gel containing 5% stem extract because F1 phenolic levels only decreased 4.65%, while F2 phenolic levels decreased 17.28% at week 8 in low-temperature storage.

#### 3.7.1. Dermatological Safety Test (Skin Irritation Evaluation)

Among 30 women volunteers, 1 participant dropped out as she was unable to attend the test on the second day. The results of the dermatological safety test showed that 25 participants had no skin lesion at both the right volar upper arm (placebo F0) and the left volar upper arm (treatment F2). Erythema was found in 1 person on the right side and 3 persons on the left side. Papule lesion was found in 2 persons on both the right and left side. The topical preparation indicated irritation of the skin if there was a primary lesion (edema) at the patch area. Moreover, all of 29 participants did not complain of itching and burning sensation in the patch area.

#### 3.7.2. Antiaging Efficacy Test

A total of 29 participants who passed the irritation test continued to take the antiaging efficacy test. However, only 28 participants took part in the study until the end because 1 participant was not present at week 2 of the examination.

**Figure 5:**
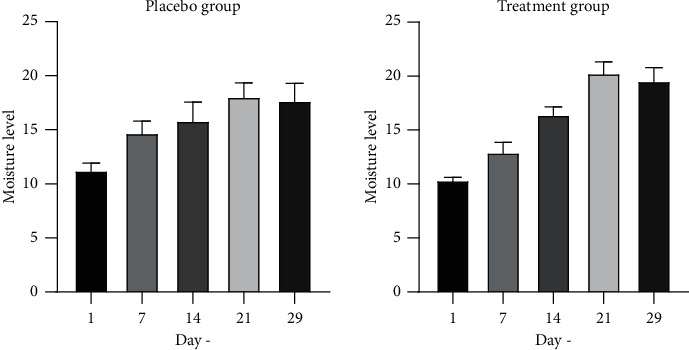
Moisture level.

The results of moisture measurement of the placebo and treatment group can be seen in Figure 5. Then, moisture analysis was conducted using one-way ANOVA and Kruskal–Wallis methods. The results obtained for the moisture of the placebo group changed significantly on day 21 (*p*=0.0024) and day 29 (*p*=0.0073). In the treatment group, the moisture also significantly changed on day 14 (*p* < 0.0001), day 21 (*p* < 0.0001), and day 29 (*p* < 0.0001). The results showed that usage of topical gel preparation, both F0 and F2, significantly affected the skin moisture level. The skin moisture level significantly improved after 21 days of using the gel base or after 14 days of using of gel with 10% *Rhus javanica* stem extract.

**Figure 6:**
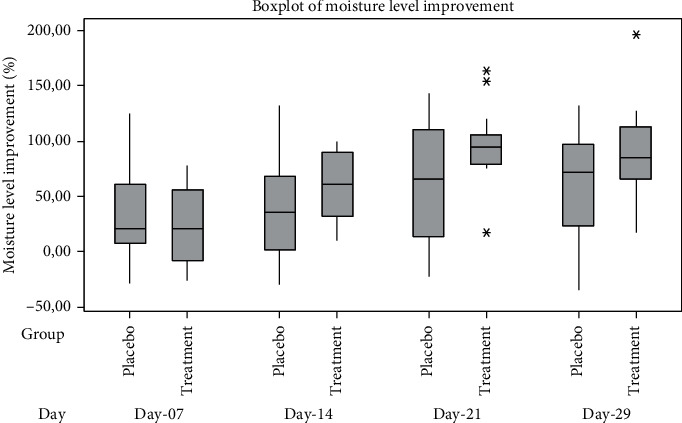
Moisture level improvement.

The improvement in the skin moisture level of the placebo and treatment group was calculated as a percent change from skin moisture level on day 1 before testing. The box plot of skin moisture level improvement of the placebo and treatment group can be seen in Figure 6. The independent *t*-test was conducted to evaluate the significant difference in skin moisture level improvement between the placebo and treatment groups. The results showed that the improvement in the skin moisture level of treatment group was not significantly higher than placebo group after 7 days of usage (*p*=0.2777) and after 14 days of usage (*p*=0.1149). However, improvement in the skin moisture level of the treatment group was significantly higher than the placebo group after 21 days of usage (*p*=0.0369) and after 29 days (*p*=0.0510). Hence, the efficacy of gel preparation with 10% *Rhus javanica* stem extract in skin moisture level improvement was significantly higher than that of gel base preparation after 21 days of topical usage.

**Figure 7:**
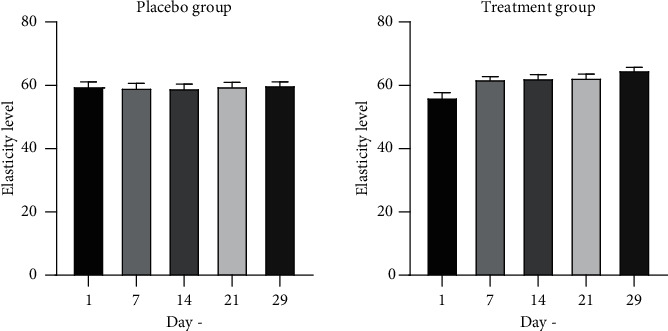
Elasticity level.

The result of elasticity measurement of placebo and treatment group can be seen in Figure 7. Elasticity analysis was also conducted using one-way ANOVA and Kruskal–Wallis methods. There was no significant change of elasticity in the placebo group. In contrast, there was a significant change of elasticity in the treatment group on day 14 (*p*=0.0485), day 21 (*p*=0.0537), and day 29 (*p*=0.0002). The results showed that usage of topical gel preparation with 10% *Rhus javanica* stem extract significantly affected the skin moisture level after 14 days of usage.

**Figure 8:**
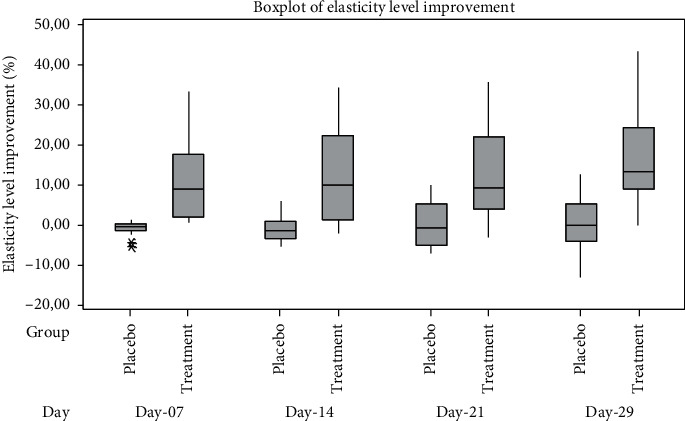
Elasticity level improvement.

The improvement in skin elasticity level of placebo and treatment group was calculated as percent change from the condition on day 1 before testing. The box plot of skin elasticity improvement of the placebo and treatment group can be seen in Figure 8. The independent *t*-test was conducted to evaluate the significant difference of skin elasticity improvement in the placebo and treatment groups. The results showed that the improvement in skin elasticity level of treatment group was significantly higher than that of placebo group after 7 days of usage (*p*=0.0002), after 14 days of usage (*p*=0.0003), after 21 days of usage (*p*=0.0009), and after 29 days of usage (*p*=0.0001). Hence, the efficacy of gel preparation with 10% *Rhus javanica* stem extract in skin elasticity level improvement was significantly higher than gel-based preparation after 7 days of topical usage.

The correlation between the in vitro antielastase activity and efficacy test was determined by the Pearson correlation test. The test analyzed the correlation of IC50 antielastase and elasticity level and also the correlation of IC50 antielastase and moisture level.

The correlation of IC50 antielastase and elasticity had a perfect correlation (-0.891). In contrast, a weak correlation occurred on the correlation of IC50 antielastase and moisture level (−0.347). The increase of moisture levels in both test groups was possible due to the influence of glycerin on the gel. The use of humectant materials such as glycerin is known to increase hydration in the stratum corneum [24]. Furthermore, HEC as gelling agent is known to have hygroscopic properties and can bind humidity in the atmosphere.

Generally, aging starts at the age of 19. Skin aging is also associated with loss of skin moisture. The primary chemical substances involved in skin moisture are hyaluronic acid and glycosaminoglycan. Both of them are able to bind and retain water molecules [25]. In some participants, the moisture level decreased randomly each week as they had a history of breakfasting, common cold, and lack of sleep.

Skin dryness is commonly caused by exposure to dry air (air conditioning room), chronic exposure to hot water, taking scrub soap daily, having a chronic illness, and side effects of drugs. However, adequate skin hydration is not enough to prevent wrinkles or other signs of aging. Many factors were affected, such as intrinsic factors, i.e., genetic, as well as extrinsic damage from sunlight and the environment. More utilities for individuals already consuming adequate fluids, the use of topical emollients will improve skin barrier function and improve aesthetics on dry skin [26].

## 4. Conclusion

In conclusion, *Rhus javanica* stem extract has antielastase activity. The research also reveals that topical gel containing 10% *Rhus javanica* stem extract has potential antielastase activity by increasing the skin moisture and enhancing skin elasticity. The optimization of formulation shall be studied further to find a good preparation.

## Figures and Tables

**Table 1:** Formulation of topical gel containing *Rhus javanica* stem extract.

Chemical substances	Percentage	
	F1 formula	F2 formula
Stem extract	5%	10%
Ethanol 96%	3%	6%
Glycerine	5%	5%
Propylene glycol	5%	5%
Propylparaben	0,02%	0,02%
Methylparaben	0,18%	0,18%
Hydroxyethyl cellulose	2%	2%
Na-EDTA	0,02%	0,02%
Distilled water	Ad 100 mL	Ad 100 mL

**Table 2:** Tardiff scale.

Reaction	Score
Normal reaction skin	0
Mild macular erithema	1
Papules	2
Edematous	3 = primary irritation
Edematous and papules	4
Vesicle and bullous eruption	5

Source [16].

**Table 3:** The elasticity and moisture parameters by skin analyzer EH 900 U.

Parameter	Value (%)				
Elasticity	Loose skin 15–35	Weak 35–50	Normal 50–65	Better 65–70	Best 70–71
*Moisture*	Dry 3–4	Aging 4–10	Normal 10–15	Higher 15–30	Shiny moist 30–65

**Table 4:** Antielastase activity of EGCG.

Sample (*μ*g/mL)	Elastase inhibition (%)	Coefficient of variation	The regression equation and correlation value	IC_50_ (*μ*g/mL)
8	28,34 ± 2,69	9,48	*Y* = 0,4408x + 24,668 *R* = 0,9958	57,43
16	32,75 ± 1,68	5,12		
32	38,38 ± 2,30	5,98		
48	43,78 ± 0,14	0,32		
64	53,66 ± 2,40	4,47		
80	60,53 ± 8,19	13,53		

**Table 5:** Antielastase activity and IC_50_ value of stem extract.

Sample (*μ*g/mL)	Elastase inhibition (%)	Coefficient of variation	The regression equation and correlation value	IC_50_ (*μ*g/mL)
100	25,40 ± 0,18	0,72	*Y* = 0,4408x + 24,668 *R* = 0,9989	245,68
150	33,76 ± 2,67	7,91		
200	41,33 ± 1,83	4,43		
250	51,56 ± 2,84	5,51		
300	59,04 ± 2,39	4,64		

**Table 6:** Chemical composition of *Rhus javanica* stem extract.

Compound	Result
Flavonoid	+
Phenol	+
Saponin	-
Tannin	-
Terpenoid	+

**Table 7:** Total ash value.

No	Extract (Gram)	Evaporating dish (Gram)	Extract + evaporating dish (after incinerating) (Gram)	Total ash value (%)	Mean + sd (%)
1	2,0012	26,2470	26,2986	2,58	2,6 ± 0,03
2	2,0005	25,9741	26,0265	2,62	

**Table 8:** Total acid insoluble ash value.

No	Extract (Gram)	Evaporating dish (Gram)	Extract + evaporating dish (after incinerating) (Gram)	Total acid insoluble ash total (%)	Mean + sd (%)
1	2,0012	26,2470	26,2473	0,017	0,02 ± 0,004
2	2,0005	25,9741	25,9746	0,023	

**Table 9:** Formulation characteristics.

Formulation		
F0 (gel based)	F1 (gel with 5% stem extract)	F2 (gel with 10% stem extract)
Color	Translucent	*Brussels brown*	*Brussels brown*
Odor	No odor	Characteristic	Characteristic
Consistence	Smooth	Smooth	Smooth
Homogeneity	Homogeneous	Homogeneous	Homogeneous
pH	6.92	4.88	4.63
Viscosity	30200 cps	30000 cps	22400 cps

**Table 10:** pH of topical gels under Stability testing conditions.

	Week 0	Week 2	Week 4	Week 6	Week 8	Week 10	Week 12
F0
4	6.92	6.53	6.64	6.52	6.39	6.40	6.51
28	6.92	6.44	6.18	6.14	5.89	5.68	5.81
40	6.92	6.34	6.12	6.10	5.80	Syneresis	Syneresis
F1
4	4.88	4.51	4.60	4.58	4.42	4.25	4.38
28	4.88	4.52	4.59	4.53	4.43	4.36	4.31
40	4.88	4.57	4.55	4.51	4.37	Syneresis	Syneresis
F2
4	4.63	4.25	4.34	4.24	4.18	4.09; 4.08	4.03
28	4.63	4.29	4.25	4.11	4.14	4.05; 4.07	3.89
40	4.63	4.26	4.26	4.10	4.10	Syneresis	Syneresis

**Table 11:** Analysis of phenolic levels in topical gels.

Formula	Phenolic levels in topical gels (%)		
	Week 0	Week 8	Week12
F1
4°C	110,59 ± 3,10	93,31 ± 2,95	118,03 ± 0,47
28°C	110,59 ± 3,10	99,45 ± 2,12	133,46 ± 0,52
40°C	110,59 ± 3,10	224,05 + 1.56	319,52 ± 35,80
F2
4°C	81,38 ± 1,44	76,73 ± 1,29	85,81 ± 2,43
28°C	81,38 ± 1,44	86,53 ± 0,88	109,82 ± 4,76
40°C	81,38 ± 1,44	191,62 ± 2,43	222,90 ± 3,43

## Data Availability

The data used to support the findings of this study are included within the article.
